# The mesoanatomy of the cortex, minimization of free energy, and generative cognition

**DOI:** 10.3389/fncom.2023.1169772

**Published:** 2023-05-12

**Authors:** James Joseph Wright, Paul David Bourke

**Affiliations:** ^1^Centre for Brain Research, and Department of Psychological Medicine, School of Medicine, University of Auckland, Auckland, New Zealand; ^2^School of Social Sciences, Faculty of Arts, Business, Law and Education, University of Western Australia, Perth, WA, Australia

**Keywords:** free energy principle, generative cognition, unlimited association, synchronous oscillation, cortical embryogenesis, cortical self-organization, neuronal homeostasis, apoptosis

## Abstract

Capacity for generativity and unlimited association is the defining characteristic of sentience, and this capacity somehow arises from neuronal self-organization in the cortex. We have previously argued that, consistent with the free energy principle, cortical development is driven by synaptic and cellular selection maximizing synchrony, with effects manifesting in a wide range of features of mesoscopic cortical anatomy. Here, we further argue that in the postnatal stage, as more structured inputs reach the cortex, the same principles of self-organization continue to operate at multitudes of local cortical sites. The unitary ultra-small world structures that emerged antenatally can represent sequences of spatiotemporal images. Local shifts of presynapses from excitatory to inhibitory cells result in the local coupling of spatial eigenmodes and the development of Markov blankets, minimizing prediction errors in each unit's interactions with surrounding neurons. In response to the superposition of inputs exchanged between cortical areas, more complicated, potentially cognitive structures are competitively selected by the merging of units and the elimination of redundant connections that result from the minimization of variational free energy and the elimination of redundant degrees of freedom. The trajectory along which free energy is minimized is shaped by interaction with sensorimotor, limbic, and brainstem mechanisms, providing a basis for creative and unlimited associative learning.

## 1. Introduction

The search for rules that account for the function of the mammalian brain, and more specifically the role of the cerebral cortex, is long-standing and central to biological neuroscience, as well as to the development of artificial intelligence (e.g., James, 1890; Freud, 1895; Sherrington, 1906, 1940; Pitts and McCullough, 1947; Hebb, 1949; Ashby, 1960; Young, 1964; Edelman, 1987; Domingos, 2015). Somehow the development and interaction of individual cortical neurons, each of which has its own metabolic necessities, lead to sentience. Two recent proposals seek to define essential features at each of the highest and lowest levels: the concept of unlimited associative learning, on the one hand (Ginsburg and Jablonska, 2019; Birch et al., 2020), and the energy homeostasis principle of neurons, on the other hand (Vergara et al., 2019). The free energy principle of Friston and colleagues offers a powerful abstract theoretical bridge between these concepts, even to the extent that a form of neuromorphic computation can be seen to emerge inevitably in all self-organizing systems (Fields et al., 2022). How this abstract principle becomes manifest at the level of conventional anatomical cellular description requires explanation. The account of mesocortical embryogenesis developed by the present authors explains aspects of observable cortical self-organization in accordance with the free energy principle, partially meeting this need. We here extend our earlier arguments to show that there is a unity between all the above ideas and that the property of unlimited association, or generativity, is implicit in our earlier account.

### 1.1. Generativity and unlimited associative learning

The concept of unlimited associative learning defines sentience as the capacity to develop very general learning, outside the bounds of Pavlovian or operant conditioning. Within the limitation of the organism's life and capacity, sentient organisms extract what they can from the information available to them, so as to make their way in the world, without being bound to the exact sequence in which subcomponents of learning are acquired and later associated. This notion follows a long discussion of conditioning vs. spontaneous mentation, expressed particularly regarding the development of speech, in the famous Chomsky–Skinner debate (Skinner, 1957; Chomsky, 1959). Currently, the emulation of generativity is of importance in the commercial development of AI [ChatGPT (OpenAI); LAMDA (Google)].

Unlimited associative learning has appeared only in some insects (honey bees), some arthropods (squids and octopuses), and vertebrates and is associated with the evolution of cortex-like structures. It is also clear that this additional capacity for learning outside of immediate stimulus and action-bound constraints is built on the underlying, more obviously reflexive, base of (in the case of mammals) the brainstem, thalamostriate, and limbic systems—subsystems with which the neocortex interacts and acts in parallel in the reception of sensory input and execution of motor commands.

Unlimited association implies the individual's creative anticipation of potential organism/environmental interactions, with exploration of these possibilities taking place “off-line” from overt behavior—yet becoming coupled with immediate sensory experience (Pavlovian conditioning) and the consequences of expressed behavior (Skinnerian conditioning) as circumstance demands—and that selection from a large set of possible behaviors must generally be those that are favorable to the organism. How these aspects are reconciled is a part of the puzzle to be solved.

We hope to show that unlimited associative learning is a natural outcome of the enormous number of neural activity states and rapid information exchanges within the cortex and that its mechanisms are reflected in observable mesoscopic cortical anatomy. Selective development of behavior from reinforced imagination is accommodated in our account, subject to the mathematical constraints of the free energy principle.

### 1.2. The free energy principle, error minimization, and synchrony

The neurodynamic and mesoscopic growth models to be subsequently considered were developed independently of the presently understood free energy principle (Wright and Kydd, 1984; Wright, 1989). Instead, our work sprang from the consideration of the dynamics of electrocortical activity as emerging from coupled stochastic oscillators, and the analogy of electrocortical steady states to thermal equilibrium. It was recently realized that this treatment conformed to the more rigorous formalism of the proper free energy principle. Our appeal to the free energy principle is more qualitative than quantitative, and our arguments are geometric rather than analytic.

The free energy principle, spearheaded by Friston (2002, 2005, 2010a,b, 2022), Friston and Ao (2012), Friston et al. (2012, 2020), Clark (2013), Buckley et al. (2017), and Constant (2021), draws parallels between laws of nature at all levels, from the principle of least action to the organization of artificial and real intelligence. A central concept is provided by Jaynes' explicit linking of the maximum entropy principle of optimum statistical information representation to the laws of thermodynamics (Jaynes, 1957), and the principle can be understood from the perspective of inference and the Bayesian brain, or neuron (Friston, 2010a,b), as dual to Jaynes' constrained maximum entropy principle (Ramstead et al., 2022; Sakthivadivel, 2022). This perspective follows from the fact that the variational free energy of inputs to any system is its negative log evidence, or marginal likelihood, so that one can interpret self-organization in neuronal systems as a form of self-evidencing (Hohwy, 2016; Palacios et al., 2017), i.e., changing in a way to maximize the marginal likelihood of its representation of all the inputs it receives from other neuronal systems. Kiebel and Friston (2011) provide some worked examples from the viewpoint of single neurons. Friston et al. (2015) provide a related formulation of pattern formation and morphogenesis with point neurons. In terms of neuronal dynamics, the minimization of free energy leads to generalized synchrony that can be read as the minimization of surprisal. In turn, this surprisal can be regarded as a prediction error, in anticipatory interactions among coupled neuronal systems. Technically, prediction errors correspond to the free energy gradients that drive neuronal dynamics. This means that neuronal dynamics are involved in the service of minimizing prediction errors and lead to generalized synchrony that—as we will see later—emerges as correlations in synchronous oscillations.

For present purposes, another way to express the idea of self-evidencing and minimized prediction error is that within any system with a boundary (a Markov blanket) through which it must interact with the surrounding environment, an open steady state must be reached, in which equal and opposite signals are continuously exchanged through the blanket, canceling each other. Here the term “Markov blanket” means that events interior and exterior to the blanket are stochastically independent apart from their mutual dependences on the blanket states. In a physical system, this is a condition required for the stability of the enclosed system and its resistance to perturbation. In informational terms, if brain states are regarded as enclosed in a Markov blanket in their interaction with environmental states, then at an asymptotic limit of learning, when learning is complete and perfectly adapted, information exchanges between the brain and environment would correspond exactly to their mutual information. Although this asymptotic limit could probably never be reached in life, it offers an important guide when applied to the cerebral cortex. Exchange of completely matched information requires that for most efficient matching there must exist a 1:1 mapping: a single topology for information representation in the external and internal worlds—a mapping of all sensory and motor interactions with the environment onto the structure of developed synaptic connectivity, with a common metric, and common dimensionality of the external and internal worlds. Synaptic connections would have developed as pathways for neural signals to pass through so that the passages of signals replicate all the ways the organism has learned to interact with the world—and, in the case of the cortex, must form this map via its interactions with intervening subcortical systems. In the following arguments, we show that the emergence of synaptic connectivity during cortical embryogenesis and later learning leads to such 1:1 maps in realistic anatomical configurations. We not only consider the cerebral cortex as itself enclosed in a Markov blanket that separates it from the brainstem and thus the external world, but we also consider small groups of neurons in the cerebral cortex as themselves becoming enclosed in Markov blankets so that there is an enclosure of blankets-within-blankets.

Variational free energy inherits its name from free energy as in thermodynamics, which is the system's internal energy minus its entropic energy. Statistically, the dual is accuracy minus complexity. These complementary perspectives on free energy are formulated in equations (1) and (2), in terms applicable to synaptic activity and plasticity, which are later used to illustrate key emergent properties.

Equation (1) is as follows:


(1) $$\begin{array}{l} F=A-C \end{array}$$

where *A* is the population sum of action potential pulse auto-correlations in a population of excitatory and inhibitory neurons within a short epoch, and *C* is the corresponding sum of pulse cross-correlations. The electrocortical system exchanges signals with the environment in analogy to a thermodynamic system exchanging heat with both a heat source (signal generation) and a heat sink (dissipation of pulses in dendrites). Consequently, *A* is analogous to internal energy and *C* is analogous to the energy associated with entropy. *F*, which is analogous to thermodynamic free energy, is continuously minimized as cortical connections become more ordered and reciprocal. Minimization must continue in relative terms, even if the number of cells is increasing over time, as is the case during neurodevelopment, or simply because of changes in cell firing rates.

In Equation (1), both pulse auto-correlations and cross-correlations including lag correlations can be given in equivalent terms as the emitted and received synaptic fluxes, φ_*ij,ji*_ between *i*−*th* and *j*−*th* cells, where the synaptic flux is the afferent pulse rate to pre-synapses weighted by synaptic gain factors. Thus


(1a) $$\begin{array}{l} C=\frac{1}{T}\overset{n/2}{\underset{ij,ji}{\sum }}\int_{0}^{T}\int_{-\infty }^{+\infty }\varphi_{ij}\left(t\right)\varphi_{ji}\left(t-\tau \right)d\tau \;dt \end{array}$$

for *n* directed flows between cells, in combinations *ij, ji* during an epoch *T*, with lead and lags, τ, and times *t* = 0 − *T*. We will consider the changes in the cross-correlation structure as development proceeds, and we will decompose cross-correlation into interactions of combinations of excitatory, *e*, or inhibitory, *i*, pairs of neurons, and mixed pairs, thus


(1b) $$\begin{array}{l} C=C_{ee}+C_{ii}+C_{ei,ie} \end{array}$$

The second representation, Equation (2), is equivalent but is stated in terms of accuracy and complexity. As learning progresses, the complexity of the information stored in the synaptic connections must increase, as must the dimension of its statistical model, obeying a time-dependent version of the Akaike Criterion (Akaike, 1974).


(2) $$\begin{array}{l} \alpha \;\left(t\right)=2lnL\;\left(k\right)-2k \end{array}$$

Here *argminα*(*k, t*) is the Akaike Criterion optimum, which is a representation of variational free energy when minimized to determine optimum *k* at each stage of learning. It is then a measure of how reproducibly neural circuits will respond to a subsequent presentation of similar inputs. The log-likelihood, *lnL* (*k*) measures how accurately the synaptic connections represent this stored information, and *k* is the number of orthogonal dimensions or degrees of freedom required to represent partial correlations among the terms summed in Equation (1a). This is one of a number of alternative decompositions of variational free energy into accuracy minus complexity (Penny, 2012). As the amount of information stored increases with learning, so must the dimension, *k*, of the most efficient descriptive model. As learning progresses and free energy is minimized, accuracy increases but under the constraint that complexity is minimized by the elimination of redundant degrees of freedom. Thus *L* (*k*) → 1, as random synaptic connections are progressively eliminated.

### 1.3. The free energy principle and the pleasure principle

The application of the free energy principle to the brain has been controversial in certain respects (Friston et al., 2012; Bruineberg et al., 2022; Mann et al., 2022). One aspect that has been widely discussed is the possible termination of learning in conditions of minimal stimulation rather than its owner's active engagement in life (Friston et al., 2012; Clark, 2013). These problems are dealt with by ascribing the organism an inherited tendency to go into circumstances that are best for it—its “preferred states,” based upon the properties of its sensory receptors—a modern-day version of Freud's pleasure principle. Because the preferred states are those with negative surprisal, the organism is seen as solving a multiple constraint satisfaction problem. How this occurs is necessarily related to the exact means by which global free energy is minimized at meso- and micro-scales.

In this article, we attempt to contribute to our understanding of these issues by showing that stable overall synaptic structures can emerge from smaller subsets of networks that are also stable and form at preliminary and intermediate stages, subject to the initial guidance of innate “preferred states.” Acquiring a capacity for generalizing further from initial learning, the organism then becomes increasingly unbound by innate predispositions. In this way, we hope to show that the operation of the free energy principle provides the key by which unlimited association emerges assisted by, but not limited to, operant and Pavlovian learning.

### 1.4. Competitive neuronal growth

The growth of neurons from their precursor forms is subject to continuing death from apoptosis of some cells and the flourishing of others with the expansion and re-arrangement of their synaptic contacts. These processes are known to be subject to the uptake of neural growth factors and the operation of a caspase execution pathway activated in those cells that undergo apoptosis (Elmore, 2007). A comparatively simple explanation for the distinction between those cells that die and those that thrive is given in the energy homeostasis principle (Vergara et al., 2019). This argues that a crucial factor is the energy economy of individual neurons and their supporting astrocytes—a criticality forced by the extreme elongation of neurons, their large surface area, and the high metabolic demands imposed by ion pumping in axons and synapses. The homeostasis principle proposes that each neuron continually self-adjusts between an extreme of high energy utilization and activity with increasingly rapid synaptogenesis and an extreme of energy resource underconsumption—the extremes bounded by negative feedback processes on the one hand and by cell death on the other hand. Neurotropic factors and the caspase execution pathway of apoptosis are intermediaries of the metabolic processes. We accept this concept as correct in principle, without comment on specific metabolic pathways, and also argue that the engagement of neurons in synchronous oscillation, thus elevating their level of metabolic activity above the baseline action potential rate, acts as a driver for synaptogenesis and survival.

The energy homeostasis principle does not in itself account for the spatial disposition of particular synapses from/to the neuron. Another part of our purpose is to explain how during the development of the cortex synaptic dispositions develop as they do, although we do not intend to imply that the energy homeostasis principle is a sufficient model of the complexity of experience-expectant and experience-dependent plasticity. A complete account would need to consider the concurrent unfolding of the genetic program and a wealth of other factors, ranging from hormone effects and stimulus characteristics up to and including societal factors (e.g., Nelson, 1999; Mateos-Aparico and Rodriguez-Moreno, 2019; Mohammend and Khalil, 2020). All of these wider influences, in our account, would act to modulate energy-dependent growth and selection by apoptosis.

### 1.5. Cortical neurodynamics

Neural field equations that are essential for the arguments that follow are given in Wright and Bourke (2021a). The development of our specific form and parameterization of neural field equations is recounted in Wright (2016), with debts to Beurle (1956), Wilson and Cowan (1972), Freeman (1975), Lopes da Silva et al. (1976), Nunez and Katznelson (1981), and Liljenstrom (1991). The equations are sufficient to model the power spectrum, frequency wavenumber content, evoked responses, synchrony, and eigenmodes of electrocortical waves (Wright and Liley, 1995, 1996; Robinson et al., 1997, 2001; Chapman et al., 2002; Rennie et al., 2002; Henderson et al., 2022). The mechanism leading to synchrony is the selective dissipation at dendritic junctions of asynchronous pre-synaptic pulses, and summation of co-incident synaptic inputs—a universal attribute of all summing junctions, including dendrites (Chapman et al., 2002). Although synchrony can arise because neurons are driven by common inputs, and non-linear phase-locking of neurons can also lead to their synchrony, widespread synchrony always arises in the neural field when coupling strengths between cells (whether via polysynaptic or monosynaptic connections) are bidirectionally symmetric, and the cells are driven by spatially diffusing white noise inputs. It is this universal mechanism of synchronous equilibrium upon which our subsequent arguments depend. Because of this mechanism, when sufficiently driven, neurons enter into synchronous oscillation. At synchronous equilibrium, all excitatory cells fire synchronously, all inhibitory cells fire synchronously, and excitatory and inhibitory cells fire in anti-phase. At lower levels of excitation, the background firing rate of most cells in the cortex for most of the time is a low Poisson distribution of action potentials. Neurons that fire synchronously and frequently are therefore driven toward more active synaptogenesis in accordance with the energy homeostasis principle.

Transfer of these dynamic properties to simplified growth simulations requires the assumption of unified fast and slow synaptic learning rules (Izhikevich and Desai, 2003) in which synaptic strength is modified on a pulse-by-pulse basis when operating upon short-term plasticity (STP) and short-term depression (STD). Slower and more permanent synaptic gain modification follows the Bienenstock–Cooper–Munro (BCM) rule, subject to slow “floating hook” negative feedback. New synaptic growth is cumulative on the fast and slow synaptic changes and is subject to decay with synaptic disuse. Consistency with the energy homeostasis principle requires that synaptic changes are competitive on all time scales.

## 2. Cortical embryogenesis and apoptosis. The primary antenatal synaptic scaffold

The account of antenatal development of the neocortex given in Wright and Bourke (2013, 2016, 2021a,b); Wright and Bourke (2022) exploits the neural and synaptic dynamics given above in such a way that growth can be modeled by a simple force-equilibrium process. This explains the pattern of connections that develop in the embryonic cortex and early in postnatal life, emphasizing lateral organization of the cortex at the millimetric scale, with no specific attention to the cellular organization in cortical layers. While developed mainly in relation to the primary visual cortical area (V1), simulation findings indicate that the model is applicable to all cortices, whether columnar or non-columnar.

### 2.1. Anatomical considerations

**Figure 1:**
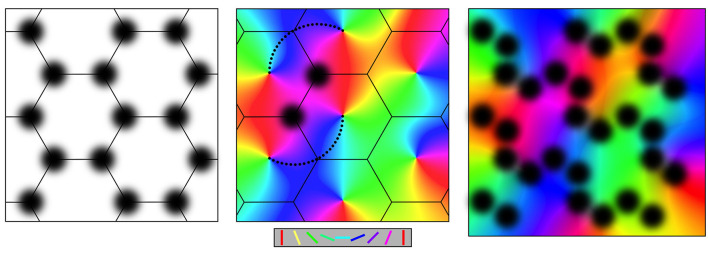
Connections at the mesocortical scale, as shown in plan view of the cortical surface. Left: Superficial patch cells in ordered array. Middle: Columnar organization of orientation preference about singularities, color-coded as shown for stimulus line orientations in the key. Like-to-like connections (small black dots) surround superficial patches and project to cells with common OP. Right: Loss of apparent structure in the non-columnar cortex, e.g.: V1 in a small animal species or non-V1 cortex. Key: Color-coded lines oriented from 0 to 180 degrees matching displayed OP.

The explanatory power of the model arises from its account of superficial patch connections and their relationship to short-axon cells surrounding the center of cortical columns in V1. Properties of orientation preference (OP), spatial frequency preference (SFP), and temporal frequency preferences (TFPs) of cortical cells and their topologies are explained as consequences of the connection geometry and conduction lags of the patch cells, as these provide contextual inputs to cortical neurons additional to direct inputs from the visual pathway. The anatomical background literature is cited in Wright and Bourke (2013, 2016), and the historical development of models for the anatomical and physiological properties is reviewed in Wright and Bourke (2022). Figure 1 summarizes the main structural features.

#### 2.1.1. Superficial patch cells

Throughout the cortex in all species, layers II and III of the cortex have aggregated patches of cell bodies of relatively long-axon pyramidal cells. The patches are approximately 50 to 300 microns in diameter, depending on the species and situation. They arise during early embryogenesis, forming synaptic connections within each patch and between patches, projecting not only to immediate neighbors but also to more distant patches. Neighboring patches that are reciprocally connected are spaced apart by about the same distance as the size of the patches and thus tile the cortical plane. In area V1, this results in a roughly hexagonal gridwork, as shown in the left-hand frame of Figure 1, or a square gridwork in ocular dominance columns.

#### 2.1.2. Cortical columns and OP

In V1, populations of short-axon cells, both excitatory and inhibitory, are enclosed by the patch cell system, forming cortical columns. The excitatory cells respond selectively to slowly moving orientated lines moving in the subject's visual field and projected by the visual pathway to the cell's position in V1, thus revealing their OP. The OP of the cells is ordered such that OP from 0 to 180 degrees circles the center of each column over 0 to 360 degrees, creating an OP singularity. Away from the singularity, OP varies continuously, and OP order within adjacent columns tends to mirror that of its neighbors, within the limits imposed by the hexagonal or square order, as shown in the middle frame of Figure 1. Continuities of OP between columns produce linear zones and saddle points.

As shown in the middle frame of Figure 1, the cells in the patch system form synaptic connections with cells in the cortical columns, with any given patch projecting to columnar cells all sharing a common OP and creating systems of “like to like” connections linked by chains of patch cells in roughly straight lines. In this way, contextual information about activity in the wider cortical field is conveyed to cells of a given OP within each column.

Cortical areas that are clearly columnar are best seen in V1 of large animal species. More generally, this ordered structure is not apparent but is blurred by overlapping patch systems, and by smearing of selective cell responses, as indicated in the right-hand frame of Figure 1. However, even at its most apparently disorganized, some vestige of the patch and column organization generally remains.

### 2.2. The model of embryogenesis

The origin of superficial patch-to-patch connectivity and “like-to-like” connections is key to the model because once these are accounted for, the organization of OP singularities, linear zones, and saddle points, in both monocular V1 and ocular dominance columns, the variation of OP with stimulus angle and speed, the interrelationship of SFP and TFP and their topological relations to OP all follow as logical consequences. No previous account has yet been able to explain more than a few of these features in a single model. In the summary below, we focus on the development of the key anatomical features as illustrated in Figure 1.

During embryogenesis, the synchronous firing of neurons protects them against apoptosis (Heck et al., 2008; Sang et al., 2021; Warm et al., 2022), as they form into small-world assemblies (Downes et al., 2012). Selection of developing neurons and synapses by apoptosis to maximize synchronous cell firing would thus shape the outcome of genetically regulated cell numbers, patterns of cell migration, and differentiation into cell phenotypes (Rakic, 2009; Geschwind and Rakic, 2013). Since synchronous oscillation is the “ground state” of equilibrium pulse exchanges among mixed excitatory and inhibitory cells, while constantly seeking equilibrium, the developing neurons are holding themselves within the domain of average firing rate that is optimal within the energy homeostasis principle.

The early selection process is followed in a population of short and long-axon excitatory intracortical cells that are mixed with short-axon inhibitory partners, linked in a very sparse network with one-to-very-many connectivity, and rich axonal/dendritic contacts, with synapses emerging at only a relatively few locations. Equilibrium requires that synaptic flux be equal in both directions between any given pair of neurons in the developing cortex or any small groups of neurons with cell bodies that are closely situated and have similar characteristics within groups. That is


(3) $$\begin{array}{l} \varphi_{ij}=\varphi_{ji} \end{array}$$

where φ_*ij,ji*_ is the exchanged pre-synaptic fluxes between *i-th* and *j-th* neurons over all pathways of connection. Competition and feedbacks inherent in synaptic learning rules lead toward bidirectional symmetry of gains along the prolific pathways, so a trend develops such that,


(4) $$\begin{array}{l} \rho_{ij}g_{ij}\epsilon_{ij}=\rho_{ji}g_{ji}\epsilon_{ji} \end{array}$$

where ρ_*ij,ji*_ is the net connectivity between the two cells because of synaptic growth, over all paths of polysynaptic connections between them, *g*_*ij,ji*_ is the synapses' net slowly consolidated synaptic gain, according to the BCM rule, and ϵ_*ij,ji*_ is their fast transient synaptic efficacy, operating within STP and STD. Each of the three factors converges toward symmetry, with some pairs of cells losing, and others increasing, reciprocal connectivity. On the fast time scale, synaptic efficacies determine the patterns of synchronous pulsing. On slower time scales, patterns of stronger synaptic coupling become established at those sites of axo-dendritic contact that favor synchrony.

Neurons unsuccessful in these competitive processes are eliminated. The selection of synapses that will increase synchrony leads to the substitution of the initial, almost entirely unidirectional excitatory synaptic links, by an increasing proportion of bidirectional monosynaptic connections. Thus, free energy, as defined in Equation (1), is progressively minimized.

**Figure 2:**
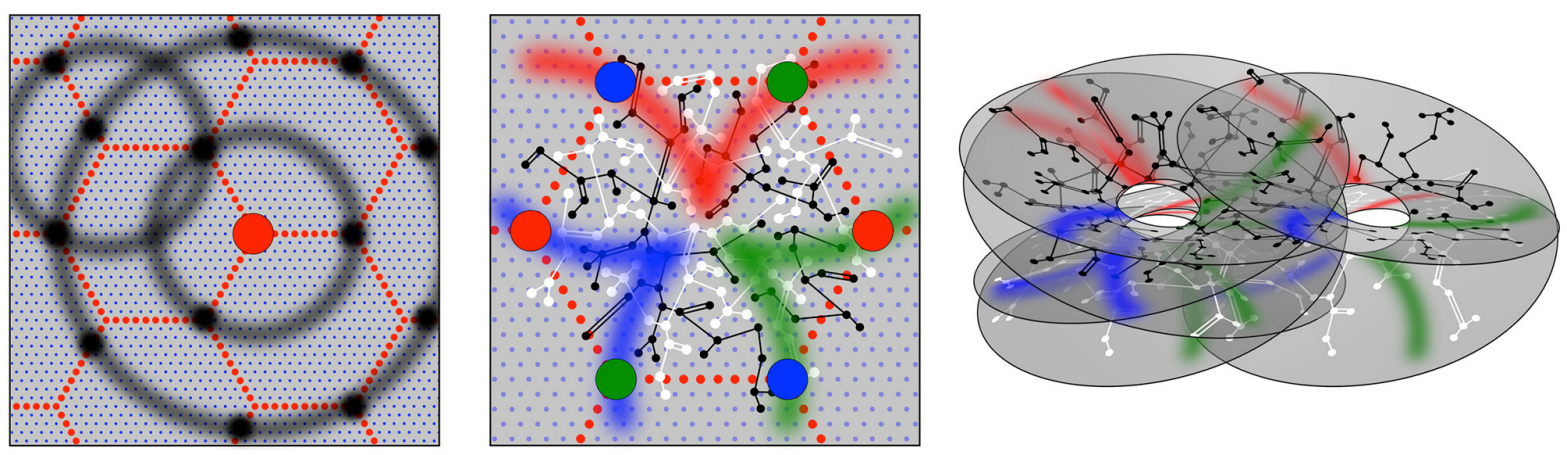
Modified from Wright and Bourke (2022). Geometric patterns of synaptic connectivity in idealized outcomes of growth simulations. Soma of short-axon local excitatory cells, forming columns, are marked by blue dots, while soma of longer axon (patch) cells are marked by small red dots, in a hexagonal array. Left: Superficial patch cells. A representative long-axon cell (large red central spot) and patch connections. Surrounding zones of potential connection with other patch cells in an ultra-small-world array have been delineated in light gray concentric circles. Dark gray patches occur where other clusters of patch cells are positioned and able to make reciprocal connections, regularly spaced, patch-to-patch. Middle: Local connectivity. Sparse short-axon cell connections have been marked in black or white, showing how interweaving networks occur. Some connections result in partial closure rather than complete independence of the interpenetrating networks. Fields of synaptic connections from patch cells to local cells are colored red, green, and blue according to their origins from diametrically opposite patch cell clusters. These oppositely placed cell groups establish synapses on interpenetrating, distinct parts of the local cell network in the pattern best maximizing synchronous resonance, and creating local maps. Right: A representation of intermingled networks of short-axon local cells conceptualized as cross-connected systems analogous to Mobius strips and viewed at an angle oblique to the cortical surface. Red, blue, and green bands indicate synaptic connections to/from the surrounding patch cell network. The degree of overlap of the small-world systems can vary from clearly columnar to blurring with the apparent absence of columnar order.

The emergence of bidirectional monosynaptic connections in patterns that maximize synchrony has effects diagrammed in Figure 2, and these reflect the basic anatomical findings shown in Figure 1.

Patch cell connections emerge because beyond a distance, *X*, from their somas, the long-axon cells have a greater population axonal density than the short-axon pyramidal cells, and vice versa, while at distance *X* axonal densities are equal for the two cell types. Linkages of the neurons into ultra-small world configuration require the long-axon cells to cluster locally, with cluster separations at distance *X*, so that they form synaptic connections with other patches, maximizing synchrony, via inter-patch connections skipping at multiples of *X* to neighboring clusters. This leads to their forming of a regular tiling of the cortex, as shown in both Figure 1 and modeled in Figure 2. Many such systems of tiled patches can form, so the patch systems can overlap, as is the case shown in the right-hand panels of Figures 1, 2.

As shown in the middle panel of Figure 2, the enclosures formed by the patch cell system are filled by short-axon local cells, forming preferential reciprocal synaptic connections at distances less than *X*. The sparsity of all connectivity means that interpenetrating networks of the short-axon cells form with sparse cross-links maximizing their total synchrony.

Between the long-axon and short-axon cell types, bidirectional monosynaptic connections emerge at the distance of separation *X*, since this is the distance at which their population axonal densities are equal. This produces a projection of the wider cortical surface (termed “the global map”) via the superficial patch cells, to neurons within neighborhood short-axon clusters (termed “the local maps”), reproducing the “like to like” connections shown in the middle frame of Figure 1. The reason OP from 0 to 180 degrees circles the singularity around all 360 degrees, as diagrammed in the middle frame of Figure 2, is as follows:

A 1:1 global map projection to each of the set of neighboring local maps must take the form


(5) $$\begin{array}{l} P\to \left\{p=\pm p^{{}^{\prime }}\frac{{\left(P-p_{0}\right)}^{n}}{|P-p_{0}{|}^{n-1}}+p_{0}\right\} \end{array}$$

where *P* is a position in the global map and *p* is a position in a local map, each designated as complex numbers; *n* determines the angular multiplication from the global to the local map; $p^{{}^{\prime }}=\sqrt{-1}k$ defines the rotation and scale of the local map; ± indicates map chirality, and *p*_0_ = *p*_0_ (1), *p*_0_ (2), *p*_0_ (3), … are the local map centers.

The reason this mapping develops is that presynapses develop from superficial patch cells to local cells (from which reciprocal connections return) in arcs of a circle of radius *X*, the arcs radiating from each map center. This requires that the projection of the global map to the local map be rotated by 90 degrees. Furthermore, a 1:1 projection of the global map to the local maps in their partially cross-connected sheets of interpenetrating networks requires *n* to take even integer values, so that, in the simplest case, angles are doubled in the projection from the global map and are thus deployed around the re–entrant-loop configuration of the local map. As a result, OP angles ranging from 0 to 180 degrees circle the singularity. The simplest case, *n* = 2, is that of projection to a single Mobius strip or to multiple cross-linked Mobius strip-like networks. Other cases representing more complicated patterns of *n* = 4, 6, 8… may also be embedded and cross-linked with each other, but will create a similarly appearing OP map.

These considerations enable us to define an elementary mesoanatomical unit as a local map and its inputs and outputs via superficial patch connections. This organization is considered elementary for the entire cortex, not restricted to V1, where its columnar form is readily apparent.

### 2.3. Antenatal cross-correlations and the dimensionality of the mesoanatomical units

Because the antenatal mesoanatomical units arise as bidirectional and symmetric monosynaptic connections emerge in a field driven by diffuse noise, in Equation (1), the corresponding cross-correlation structure has terms with maxima at zero-lag for excitatory–excitatory and inhibitory–inhibitory pairs, and maxima at the lag of the reversed phases of the excitatory–inhibitory pairs, as these maxima correspond to zero-lag synchronous equilibrium. In Equation (2), the mesoanatomical unit could be specified by a model of *k* = 3 dimension, since there is continuous rotational symmetry about the OP singularity, with a depth dimension introduced by Mobius strip-like folding of the local map. This folding disambiguates the identity of distance from the local map and the associated time lag.

## 3. Postnatal information storage on the antenatal scaffold

### 3.1. Postnatal spatiotemporal maps and spatial and temporal frequency preferences

The mesoanatomical units offer a scaffold upon which spatiotemporal images and sequences can be represented. Because antenatal connections reflect the general declining synchrony-vs.-distance relationship among cortical neurons and are structured in three dimensions, this approximates the same topological order of generally declining cross-correlation-vs.-distance relationships of the sensory surface in space and time. The antenatal map thus provides a scaffold of a sufficient dimension for the representation of stimulus objects moving in two spatial dimensions and time.

As structured inputs arrive from cortical input pathways, they create patterns of active cell firing, *O* (*P, t*), and these activity patterns are relayed via patch cells to positions {*p*} in many local maps. Because they are transmitted with conduction delays, they then give rise to activity patterns, {*o*}, within each of the local maps,


(6) $$\begin{array}{l} O\left(P,t\right)\to \left\{o\left(p,t+\frac{\left|P-p\right|}{\nu }\right)\right\} \end{array}$$

where ν is the velocity of signal conduction.

**Figure 3:**
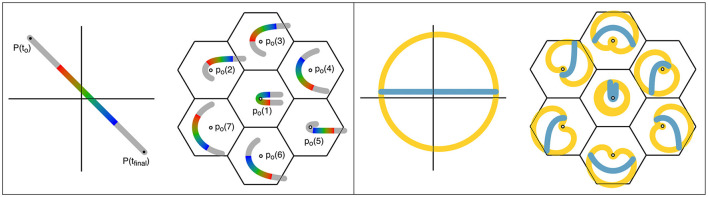
Projections from global-to-local fields in the cortex, resulting in spatiotemporal representations. **Left**: Projection of a moving point in the sensory field to the cortex. The image transit in the global field from *P* (*t*_0_) to *P* (*t*_*final*_) is marked in the colors of the spectrum, to indicate both cortical position and time of transit. Lateral relay via patch cells transfers the input to a cluster of small-world local map ensembles of cells (columns), centered in the same reference frame as the moving stimulus. All the local neurons activated within the brief epoch, marked in the appropriate color for their signal input from the global map, can enter into synchrony, thus creating a representation of stimulus position and movement within the epoch. **Right**: The same representation of global and local maps used on the left is applied. Positions in the global cortical field approximately circumferential to the reference origin are colored amber, and a line passing near the origin is colored blue. Projections of the circumferential and radial lines within the local maps retain their approximately circumferential and radial relations, and correspond to neurons with high space- and temporal frequency, and low space- and temporal frequency preferences, respectively.

Figure 3 shows this signal transfer diagrammatically. A single point of stimulus input at the global level, varying in position with time, becomes mapped to multiple concurrently pulsing neurons. Particularly if the inputs to these cells arrive in approximate synchrony, the interaction of the excited neurons will lead to the cells exchanging signals and, as they seek equilibrium, entering synchrony as a whole, by the general mechanism discussed in Section 1.5. Each of the concurrently active neurons has received information about the moving input at different positions and times. The synchronous set of neurons thus contains information about the average position, velocity, and acceleration of the moving point, within a short epoch—the epoch defined by the duration of sustained synchrony. These synaptic assemblies of cells induced to fire in synchrony are spatiotemporal images.^1^ Because the inputs are moving in two spatial dimensions and time, they can be represented on the three-dimensional space of the antenatal mesoanatomical units, but the break in connection symmetry as new synaptic connections are induced by co-synchrony requires an increase in model dimension. A series of images of this sort can be linked together by unidirectional connections to form assemblies of moving images, and at points of overlap, further bilateral connections can store partial correlations of separate images. These considerations foreshadow a postnatal increase in the dimension of the stored information.

As shown in Figure 3 and further illustrating the postnatal increase in dimension, structured postnatal inputs bring about a systematic reorganization of synaptic connectivity within the short-axons cells of the local maps, breaking the antenatal rotational symmetry (Wright and Bourke, 2022). Signals originating from global positions circumferentially arranged around the center of a local map generate circumferential connectivity within the local map, while those radial to the local map center reinforce the antenatal radial “like to like” connectivity. The response characteristics, and the anatomical distributions, of high- and low-space frequency preferences (HFSPs and LFSPs), and temporal frequency preferences (TFPs) of V1 neurons can thus be explained—the essential reason being that signals generated concurrently in circumferential positions in the global map relay to the local map with equal, or near-equal delays, generating a relatively high frequency and synchronous signal in the local cells. Conversely, signals generated concurrently at positions oriented radially in the global-to-local projection arrive with different delays and therefore generate activity at a lower frequency (Similar considerations also explain the variation of OP with stimulus velocity and angle of attack.). This leads to the generation of zones of connected local cells that respond to different input frequencies, i.e., to the creation of separate synchronous eigenmodes.

### 3.2. Maps in multiple cortical areas and motor outputs

The bidirectional connections formed in the global-to-local maps can be regarded as having a “forward” sensory relay function, as in Equation (5), but also a “backward”, motor-efferent function, as indicated by signals following connections that are the inverse of Equation (5), given by


(7) $$\begin{array}{l} P\leftarrow \pm \frac{1}{p^{{}^{\prime }}}{\left(p-p_{0}\right)}^{\frac{1}{n}}|p-p_{0}{|}^{n-1}-p_{0} \end{array}$$

Extending this organizational principle to the motor cortices, the output of motor commands follows the same form as equation (6), but in reverse—that is


(8) $$\begin{array}{l} M\left(P,t\right)\leftarrow \left\{o\left(p,t-\frac{\left|P-p\right|}{\nu }\right)\right\} \end{array}$$

where *M* (*P, t*) is the time-ordered sequence of efferent motor commands.

Signal transmission through the cortex does not, of course, simply pass directly from sensory to motor systems, but is mediated additionally via complex exchanges within association areas. How, by synchrony matching between cortical areas, information stored and manipulated at a mesoscopic scale may contribute to cortical computation, has been discussed in Wright and Bourke (2021a,b). From these exchanges, “unlimited association” must be generated. But how? We next suggest that it is in the backward and forward exchange of signals between patch cells and local maps, under the minimization of prediction error, that an important part of the solution can be found.

## 4. Postnatal modifications of connectivity under structured inputs

### 4.1. Stability approaching asymptotic limit of learning and eigenmode coupling

If the asymptotic limit of learning is to be continuously approached in the face of ongoing perturbation, the free energy principle requires that the difference in exchanged signals must be progressively minimized to zero at critical parts of the network—all those higher and lower in a hierarchy of signal flow.

In addition to the flows of signals into, from, and between cortical areas, which take place via topographic projections, we need to consider flows of signals laterally across the cortical surface in the connections between patch cells and short-axon cells described in Equations (5) and (7). At the learning asymptote, the two-way flow must cancel the flow from lower to higher in the hierarchy. This requires the generation within the local map network of patterns of activity that replicate in anticipation the pattern of activity that will next arrive in the global map—the reverse of arrivals described by Equation (6)—so that


(9) $$\begin{array}{l} \left\{o\left(p,t-\frac{\left|P-p\right|}{\nu }\right)\right\}\to O\left(P,t\right). \end{array}$$

The generation of this patterned activity can be described as the action of coupled eigenmodes, i.e., fields of synchronous activity that interact in some way with each other to produce the required time-varying patterns. More complicated cross-correlation terms must then appear in Equations 1, 1a, and 1b.

There must also be certain cells where the forward and backward flows converge to cancel, so only for these cells, where {ǒ} ∈ {*o*} and {ǒ} ∈ {*O*}


(10) $$\begin{array}{l} \left\{ǒ\;\left(p,t\right)\right\}↔ǒ\;\left(P,t\right). \end{array}$$

This set of cells acts as a Markov blanket between the global field and each of the local maps.

These considerations raise the question of how synaptic connectivity in the neural field must be modified to serve the functions of eigenmode coupling and the formation of mesoscale Markov blankets.

### 4.2. Changes in synaptic flux and connectivity

To facilitate the consideration of the coupling of spatial eigenmodes, the neural field can be represented as stochastic oscillators, each oscillator being a small group of interacting excitatory and inhibitory cells, and each group linked at longer range by excitatory cells (Wright, 1989), where φ_*i,j*_ are the excitatory synaptic fluxes in the small groups. where φ_*i*_ is the excitatory synaptic flux generated in one of these small groups, φ_*i,j*_ is the excitatory synaptic fluxes in the small groups, and {*D*_*i*_, *N*_*i*_, *K*_*ij*_} are time-varying stochastic parameters analogous to damping factors, natural frequencies, and coupling strengths of harmonic oscillators, then


(11) $$\begin{array}{l} {\ddot{\varphi }}_{i}+D_{i}{\dot{\varphi }}_{i}+N_{i}^{2}\varphi_{i}=\underset{j}{\sum }K_{ij}\varphi_{j}. \end{array}$$

When driven by the diffuse white noise of wide bandwidth *W*, the field has eigenmodes with frequencies, *M*_*i*_, and damping factors $D_{i}$. If total energy is *U*, and *U*_*i*_ is the energy of the *i*−*th* mode, then the entropy of the *i*−*th* mode is


(12) $$\begin{array}{l} \sigma_{i}=\left(\frac{U_{i}}{U}\right)\ln\left(\frac{U_{i}}{U}\right). \end{array}$$

Applying these terms to the Gibbs equation for free energy, we get


(13) $$\begin{array}{l} F=U-\tau \sum \sigma_{i} \end{array}$$

where *F* is again free energy and τ is a constant that scales the average entropic energy per mode. This is the thermodynamic equivalent to Equations (1) and (2). Free energy reaches the absolute minimum when energy is equipartitioned between resonant modes, and entropy is thus maximized. Equipartition of energy requires all $D_{i}$ are equal.^2^

**Figure 4:**
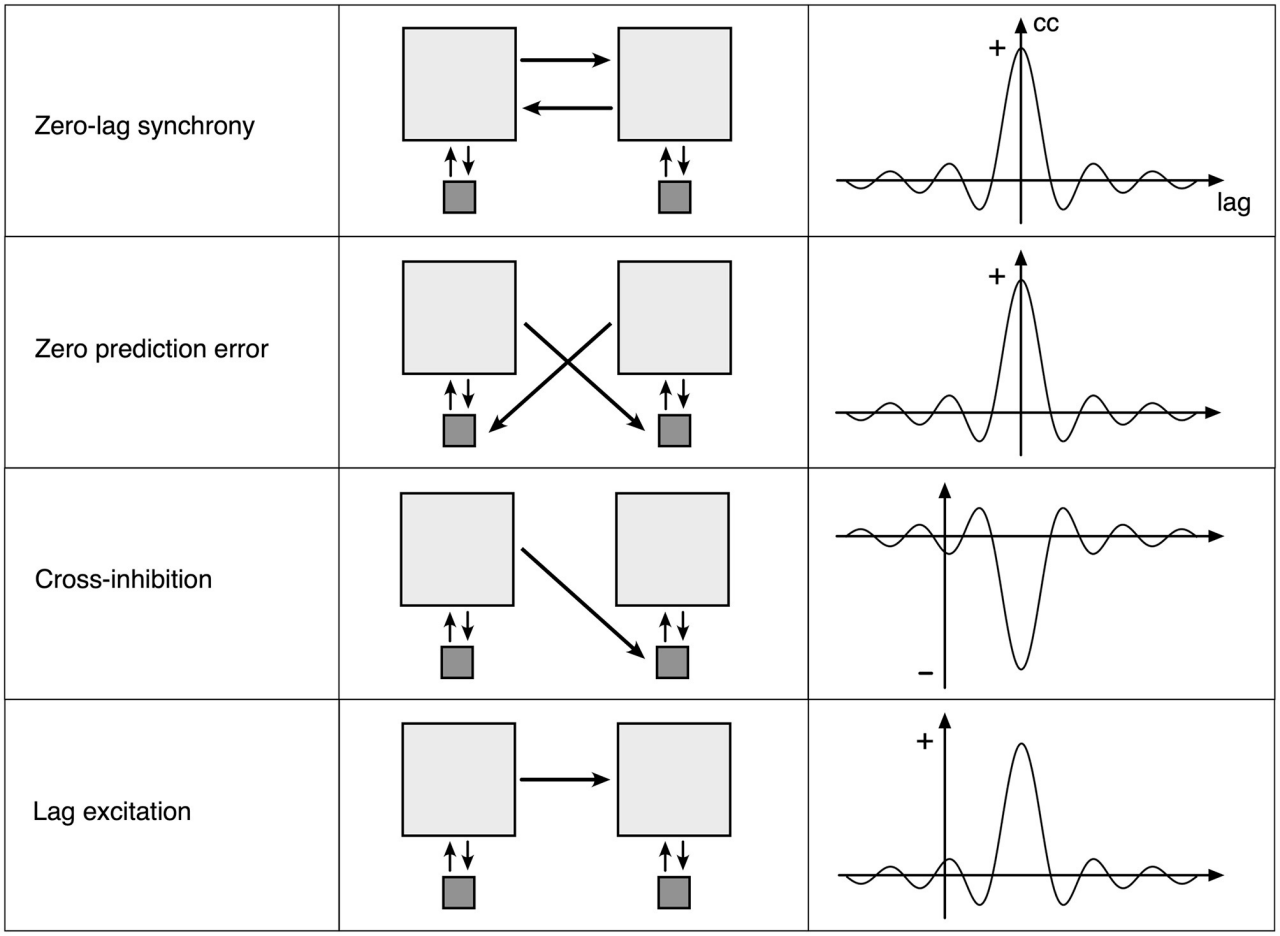
Four elementary types of synaptic flux exchange in the perturbed neural field. **Left** column: a consequence of the type of flux exchange. **Middle** column: Light gray squares indicate small groups of excitatory neurons, and dark squares indicate small groups of inhibitory neurons. Arrows indicate the strength and direction of synaptic flux. **Right** column: an approximation of induced cross-correlation of excitatory pulse densities.

How spatial eigenmode coupling and construction of Markov blankets can arise follows simply as departures of synaptic connectivity from the antenatal near-equilibrium case. Figure 4 shows the four ways that changes in synaptic flux (and subsequent synaptic consolidation) could occur.

The top row represents the symmetric exchange of synaptic flux between groups of excitatory cells. Excitatory flux exchanges are in phase with opposite excitatory cells, so that zero-lag synchronous oscillation is sustained. Flux dissipation is minimal. This corresponds to the antenatal state, and with a subsequent strengthening of connections, to the postnatal formation of spatial eigenmodes.

The second row indicates that the symmetric exchange of excitatory synaptic flux is directed to opposite inhibitory cells, so that excitatory flux is received in anti-phase to the opposite inhibitory pulse activity, thus suppressing oscillation if sufficiently strong. Flux dissipation is maximal, and perturbation is minimized. Each excitatory group cancels activity in the other if each group has predicted the other with zero error, in which case they also remain in zero-lag synchrony. The zero-error case can be decomposed into twin unidirectional flows of the third type, cross-inhibition.

The third row represents an asymmetric exchange of excitatory synaptic flux directed to opposite inhibitory cells so that one excitatory group can suppress oscillation in the other, producing cross-damping of synchronous eigenmodes.

The fourth row indicates an asymmetric exchange of excitatory flux between excitatory groups, with conduction delay, permitting self-exciting chains and the “winnerless synaptic competition” (Rabinovich et al., 2008) required for heteroclinic neural networks.

The second, third, and fourth rows introduce increasingly complicated lag correlation terms into *C* of Equations 1, 1a, and 1b and increasing *k* in Equation (2). The next section describes how such connection modifications can be embodied within mesoscopic anatomy.

### 4.3. The anatomical embodiment of postnatal synaptic evolution

**Figure 5:**
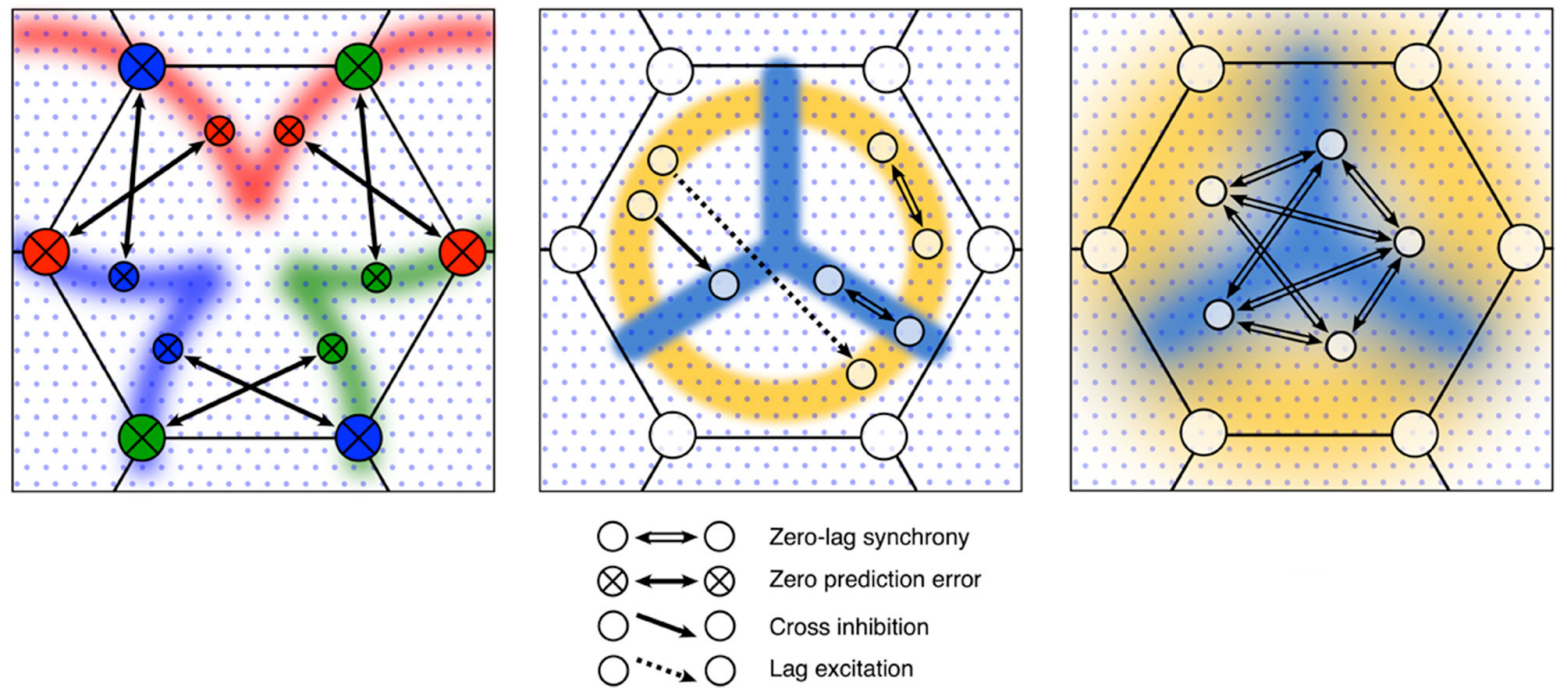
Evolution of synaptic connections in the antenatal scaffold following the introduction of structured postnatal inputs. **Left**: Zero-error connections develop between the patch and local cells. **Middle**: Radial and circumferential zones, shown in amber and cyan, emerge as local cells become tuned to low temporal frequency, and high temporal frequency preferences, respectively. Anisotropic unidirectional monosynaptic cross-inhibition and lag-excitation couplings develop, mediating the dynamics of coupled spatial eigenmodes. **Right**: Local response preferences are smoothed by local interactions—shown as increased continuity of the blue and amber zones. Bidirectional monosynaptic connections mediating increased and interpolated patterns of synchrony increase as random connections are eliminated.

Figure 5 shows changes from antenatal connectivity toward meeting postnatal conditions. It is notable that as these synaptic changes can be induced because excitatory neurons are always intimately entangled with inhibitory cells, all four types involve only local shifts in synaptic connections, and could therefore take place with relatively high metabolic efficiency. This helps explain how the energy homeostasis of entire neurons can be closely linked to the generation of specific synapses, since small local changes in connectivity, complying with the STD, STP, and BCM rules at the microscale, can have major effects on synchrony and thus metabolic demand throughout the neuron soma.

Mesoanatomical units can become stabilized by the development of zero-error connectivity at patch cell connections to local cells, replacing antenatal zero-lag synchronous connections, thus moving toward the construction of a Markov blanket. Concurrently, cross-inhibition and lag-excitation connections can develop within each local map as temporal and spatial frequency preferences become tuned, giving rise to groups of excitatory cells arranged in contrasting, anisotropic, orthogonal systems that, by interacting through common inhibitory cell neighbors, couple spatial eigenmodes, while lag-excitation orchestrates time sequencing of the eigenmode switching. Cross-correlations must now involve more complicated lag terms, with an increasing dimension of information storage. Corollary to these adaptations, all local excitatory cells can further adapt their connections to maximize their co-synchrony, eliminating some of the noisiness of the antenatal connectivity that developed in the presence of diffuse noisy inputs, raising model likelihood to match increasing model dimension, and in so doing actively interpolate values by smoothing of surrounding connectivity by joint synchrony.

As these changes take place, the mesoanatomical unit continues to respond synchronously to diffuse noise in the global field—so that unless specifically engaged by structured inputs the unit sends only low information signals to other units. The asymptotic limit of learning can be approached when zero-error couplings increasingly act to stabilize activity—but each unit will become perturbed when local and global signals are asymmetric so that inputs convey high surprisal.

## 5. Emergent unlimited associations and generativity

### 5.1. Interareal exchanges and the generation of possibilities

The overlap of many mesoanatomical units permits their merging into larger and larger ensembles, always in accordance with the minimizations of Equations (1) and (2). These larger systems are referred to as “assemblies” in Hebb's terminology for “cell assemblies.” This can develop by a process of enclosure, in which smaller component assemblies become ringed with enclosing chains of zero-error connections, while the enclosed units can furnish ever more complicated patterns of eigenmode coupling. This can take place by the generation of zero-error types of connection between the chains of patch cells.

Transmissions to and from the growing assemblies can propagate rapidly throughout the cortex, via cortico-cortical fibers, resulting in a combinatorial explosion of image superpositions from cortical areas *P*_*X*_, *P*_*Y*_, *P*_*Z*_, to any given area, *P*_*A*_


(14) $$\begin{array}{l} P_{X}+P_{Y}+P_{Z}+\ldots \to P_{A}. \end{array}$$

With subsequent lateral propagation into local maps, we get


(15) $$\begin{array}{l} P_{A}\to \left\{p_{A}=\pm p^{{}^{\prime }}\frac{{\left(P_{A}-p_{0}\right)}^{n}}{|P_{A}-p_{0}{|}^{n-1}}+p_{0}\right\}. \end{array}$$

Most assemblies and sequences will be transient, but those that minimize free energy will persist, progressing through the three separate time scales of connection formation of Equation (4), ultimately resulting in fusions cortex-wide.

### 5.2. Subsequent evolutionary selection of assemblies and innovative outcomes

We can now turn consideration toward the role of preferred states in the application of the free energy principle. In a recent review, Tucker and Luu (2023) describe the functional significance of the anatomical studies of Barbas (1986), Barbas and Rempel-Clower (1997), and Garcia-Cabezas et al. (2019) revealing the pathways of migration of cells from the embryogenic paleo- and archi-cortex that generate the dorsal and ventral divisions of the neocortex and form a central core of paleo- and archi-cortex with radial neocortical areas. This study emphasizes the rich interactions of all neocortical areas with limbic, sensory, and motor systems, including attentional mechanisms, and Tucker and Lu relate this structure to differences in dorsal and ventral systems of the role of surprisal and prediction error minimization, depending on the level in the hierarchy of information flow. It is within such a global system that we envisage the creation and merging of assemblies taking place.

**Figure 6:**
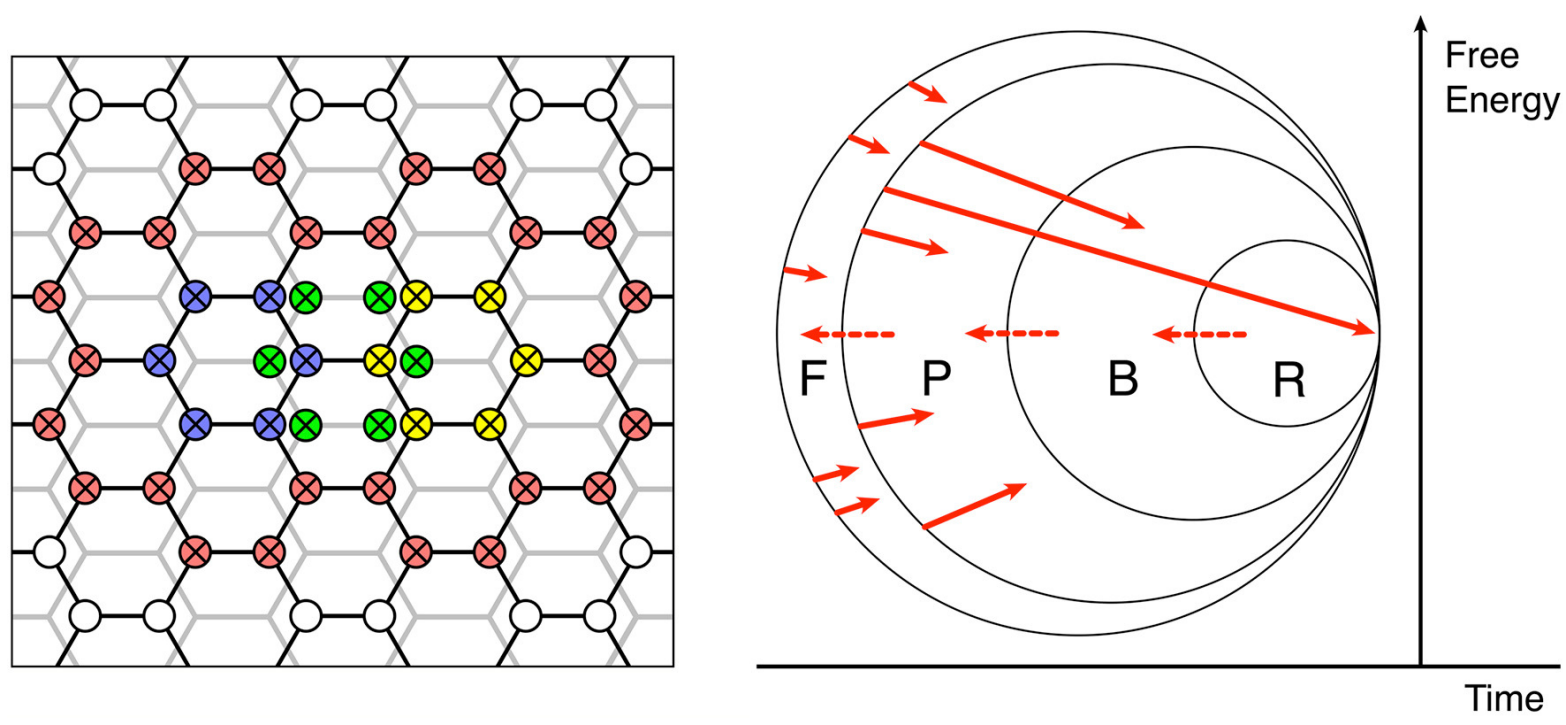
The continuing evolution of synaptic connections into a large-scale functional organization. **Left**: Enclosure and interdigitation of progressively greater cortical areas by zero-error connections—red zero-error connections enclose smaller units in overlapping networks of previously formed zero-error enclosures, shown in blue, yellow, and green. **Right**: Stages of selection or competitive dissipation, defined by the subsets F, P, B, and R.

The left side of Figure 6 shows how the initial assemblies can undergo merging into larger conglomerates, by enclosing a number of assemblies within larger rings of zero-error connections among patch cells.

The right side of Figure 6 represents, as a Venn diagram, how the self-assembly of synaptic connectivity, excited by superpositions of inputs can ultimately result in collective assemblies capable of generative cognition.

The right-hand facing arrows show by their origins and arrowheads the initiation of assemblies and their termination within the subsets F, P, B, and R.

Assemblies arising in F (failed) comprise the following:

- all those that do not meet the demand for organized zero-error connections and coupled eigenmodes satisfying the matching of forward and backward signals (Equation 10) required for continuing self-stabilization by gradient descent in free energy, or
- cannot link with others into configurations surrounded by zero-error connections also satisfying equation (10) at a larger scale, or
- require increasing external energy to achieve transition into configurations meeting (a) or (b) when such inputs are not available.

All these must dissipate under competitive pressure from other assemblies.

Assemblies arising in P (possible) are those that meet the primary requirements not attained by those in set F. However, they too will dissipate if they are unable to form associations based on sensory preferred states, with assemblies falling in the B (behavioral) set. These components potentially capable of generating actions, either overtly or through internal adjustments of attention that acquire linkage with motor expression and overt behavior, without which they, too, will dissipate under competitive pressures due to a lack of a path for forward evolution. Innately generated actions must include escape from averse stimuli and approach to the pleasurable and are thus the motor equivalent of sensory preferred states.

But to persist toward permanency, assemblies must further gain access to R (reinforcement). That is, they must produce behaviors (including cognitive operations) that are consistent with innate limbic and subcortical components in the reinforcement of behavior.

Reinforcement pathways that can be artificially activated during intracranial self-stimulation (Olds and Milner, 1954) and are mediated by dopaminergic and related pathways (Wightnan and Robinson, 2002) exert diffuse effects cortex-wide (Wright, 1973). They are concerned with cortical activation and the driving of motor behavior, and also modulate synaptic consolidation (Pawlack and Kerr, 2008; Shindou et al., 2019), thus strengthening all synapses recently active, even in widely distributed assemblies. These R-actions, too, can be considered a part of the “preferred states” ensemble of the organism, but are not simply equivalent to the action of hard-wired sensory and pain receptors. They introduce the capacity for internally generated designation of current sensory states as “preferred,” by designating certain states as more salient than others. As assemblies are added to the inner set by reaching R, they can expand the membership of the R, B, and P sets (indicated by the dotted right-to-left red arrows at the boundaries of the sets), increasing the available assemblies upon which further expansion can take place.

Overall, then, the system can find structure within its inner world as it moves toward the learning asymptote and has an unbounded capacity for innovation—unlimited association—within its lifetime.

## 6. Conclusion

The same relatively simple model of neuronal and synaptic dynamics has been applied throughout the antenatal and postnatal stages of development. The wide range of features of mesocortical anatomy that are accounted for have been mentioned in preceding sections, and the free energy principle is complied with at all stages and scales. We have specified the stages of outcome in a motor and reinforcement-related evolution and showed how this may lead to an unlimited association or generativity.

The development of a map of the external world goes through stages from simple to more complex. The early embryonic self-organization maps the general decline of cross-correlation in space and time of the external world onto the general decline of cross-correlation of synchrony among cortical neurons, forming a scaffold for the representation of elementary spatiotemporal images. The scaffold acts as a position holder so that as further cross-connections are added, the partial correlations of stimuli (and actions) in space and time maintain mutual consistency. As more highly structured signals reach the cortex in postnatal life, the initial framework of simple synchronous connections provides a starting point, from which further modifications of synaptic connectivity can take place by simple local transfers of synaptic connectivity in a metabolically efficient fashion and in accordance with the neurons' homeostatic principle. Attaining and sustaining a stable state is possible both locally and globally via gradient descent in free energy. Implicit in this self-stabilization is also the retention of sensitivity to surprise required in realistic learning short of the theoretical asymptote, as is the minimizing of unwanted cross-talk among trained units.

The elimination of random connections and ongoing smoothing of connections by synchrony acts as an interpolative and anticipatory process, offering the best prediction of further modifications that may be required as learning progresses.

Further quantitative development of this concept in rigorous terms of the free energy principle is possible, but it is beyond our scope. Such a quantitative approach might consider, for example, the provision of activation energy by driving from subcortical activation and attention that might bring about fusion of initially rather unlikely large assemblies—an analog for sustained thought. Replication in the simulation of more detailed learning, taking advantage of the properties of the scaffold and the relatively simple and restricted form of local synaptic updating, seems not beyond practicability.

Our proposal must face the test of integration with detailed synaptic and cellular physiology. Similar synaptic modifications minimizing prediction errors, detecting surprisal, and co-ordinating interactions can operate equally well via the topographic interareal (cortico-cortical) projections, so an integration between mesoscale and whole brain dynamics seems possible.

## Data availability statement

The original contributions presented in the study are included in the article/supplementary material, further inquiries can be directed to the corresponding author.

## Author contributions

JW devised and wrote the manuscript. PB was responsible for software and graphics. Both authors contributed to the article and approved the submitted version.

## Footnotes

^1^Any single spatiotemporal image need only be an incomplete representation of stimulus properties. If the points and times in *O*(*P, t*) are widely separated, the image will covey information mainly about the “where” and “when” of the stimulus. If the points are closely located, then the emphasis will be on stimulus details – of “what” it is. This corresponds to receptor field tuning of neurons in the dorsal and ventral neocortical systems respectively (Tucker and Luu, 2023).

^2^The approach to equality of $D_{i}$ in alert electrocorticogram has been shown using autocorrelation analysis (Wright et al., 1990).
